# *TP53* DNA Binding Domain Mutations Predict Progression-Free Survival of Bevacizumab Therapy in Metastatic Colorectal Cancer

**DOI:** 10.3390/cancers11081079

**Published:** 2019-07-30

**Authors:** Hung-Chih Hsu, Jeng-Fu You, Shu-Jen Chen, Hua-Chien Chen, Chien-Yuh Yeh, Wen-Sy Tsai, Hsin-Yuan Hung, Tsai-Sheng Yang, Nina Lapke, Kien Thiam Tan

**Affiliations:** 1 Division of Hematology-Oncology, Chang Gung Memorial Hospital at Linkou, Taoyuan City 333, Taiwan; 2 College of Medicine, Chang Gung University, Taoyuan City 333, Taiwan; 3 Division of Colon and Rectal Surgery, Chang Gung Memorial Hospital at Linkou, Taoyuan City 333, Taiwan; 4 Chief Scientific Officer, ACT Genomics Co. Ltd., 3F., No.345, Xinhu 2nd Rd., Neihu Dist, Taipei City 114, Taiwan; 5 Chief Executive Officer, ACT Genomics Co. Ltd., 3F., No.345, Xinhu 2nd Rd., Neihu Dist, Taipei City 114, Taiwan; 6 Department of Medical Informatic, ACT Genomics Co. Ltd., 3F., No.345, Xinhu 2nd Rd., Neihu Dist, Taipei City 114, Taiwan

**Keywords:** metastatic colorectal cancer, bevacizumab therapy, next-generation sequencing, progression-free survival, *TP53* DNA binding domain mutation

## Abstract

(1) Background: Bevacizumab-based regimens are a standard treatment for metastatic colorectal cancer (mCRC) patients, however meaningful clinical biomarkers for treatment benefit remain scarce. (2) Methods: Tumor samples from 36 mCRC patients treated with bevacizumab-based chemotherapy underwent comprehensive genomic profiling. Alterations in frequently altered genes and important signaling pathways were correlated with progression-free survival (PFS). (3) Results: Overall genetic alteration analysis of investigated genes and pathways did not identify promising new predictors of PFS. However, when considering mutation subtypes, *TP53* DNA binding domain (DBD) missense mutations were associated with prolonged PFS (HR, 0.41; 95% CI, 0.13−0.65; *p* = 0.005). In contrast, *TP53* truncating mutations were associated with short PFS (HR, 2.95; 95% CI, 1.45−27.50; *p* = 0.017). Importantly, neither *TP53* mutation subtype was associated with overall response rate. In multivariate analysis, *TP53* DBD missense mutations remained an independent PFS predictor (HR, 0.31; 95% CI, 0.13–0.77; *p* = 0.011). The other genetic factor independently associated with PFS were *PTPRT/PTPRD* deleterious alterations, which we previously identified in a screen for biomarkers of bevacizumab response. (4) Conclusions: *TP53* DBD missense mutations may predict prolonged PFS in mCRC patients treated with bevacizumab-based therapy. Analyses of *TP53* mutations as clinical biomarkers should take the biological impact of different mutation subtypes into consideration to improve patient stratification.

## 1. Introduction

Colorectal cancer (CRC) represents one of the most prevalent cancer types in both sexes [1]. Although screening is becoming more common, more than 20% of CRC patients are diagnosed with metastatic disease [2]. In addition, 30% of early stage CRC patients progress to recurrent or metastatic disease [3], and recurrent or metastatic CRC (mCRC) patients have unfavorable outcomes.

Bevacizumab is an antibody binding specifically to the circulating vascular endothelial growth factor A (VEGF-A) [4] and inhibiting tumor angiogenesis. It has been widely used in combination with chemotherapeutic regimens in various cancers, including mCRC, and prolongs mCRC patient survival in combination with chemotherapeutic agents [5,6,7,8]. However, predictive biomarkers for bevacizumab in mCRC are not yet available as guidance for clinical practice.

Suggested genetic markers for bevacizumab benefit include copy number variations [9]. Mutational markers have also been investigated, but previous studies have mainly focused on the analysis of overall mutations in pre-defined key genes, such as *TP53* and *KRAS*, with limited success [10,11]. However, associations could have been missed, since genetic markers of drug benefit could include multiple genes of a certain cancer pathway, since copy number alterations of genes of interest as biomarkers may have been overlooked. On the other hand, mutational subtypes of certain genes may be functionally different and therefore differently impact a cancer treatment outcome, as observed for *TP53* mutation subtypes [12].

In a screen for biomarkers of bevacizumab response by comprehensive genomic profiling, we recently identified deleterious mutations and copy loss in the genes *PTPRT* and *PTPRD*, phosphatases of the JAK/STAT pathway, as biomarkers of outcome [13]. However, since it has been shown that benefit from bevacizumab can be observed independent of the treatment response [14], we further aimed to identify markers that may be associated with progression-free survival (PFS) in our cohort, but might not have been identified before due to a lack of association with response. For correlation with PFS, we analyzed sequence and copy number variants detected by next-generation sequencing of tumor samples from 36 bevacizumab-treated patients. We analyzed a variety of important cancer signaling pathways, both on a whole pathway and single-gene level. Furthermore, the possibility of a differential association of mutational subtypes in frequently altered genes with PFS was taken into consideration.

## 2. Results

### 2.1. Patients

A total of 36 patients with metastatic colorectal cancer were enrolled, and patients have been characterized in a previous publication from the same study [13]. In brief, all patients were treated with bevacizumab plus chemotherapy with FOLFIRI (irinotecan and infusional 5-fluorouracil with leucovorin) [15]. Patients showed a relatively balanced distribution regarding sex (58% male, n = 21 and 42% female, n = 15), metastatic pattern (53% synchronous, n = 19 and 47% metachronous, n = 17) and primary tumor site (58% colon, n = 21 and 42% rectum, n = 15). The median age was 60 years (range: 33–87 years), and the proportion of patients with more than one metastatic site was 58% (n = 21). The majority of patients harbored low-grade tumors (92%, n = 33) and received bevacizumab plus chemotherapy as a first-line treatment (89%, n = 32). All patients had a microsatellite stable (MSS) disease.

### 2.2. Genetic Alterations in Oncogenic Pathways and Progression-Free Survival in Bevacizumab-Treated Patients

**Figure 1:**
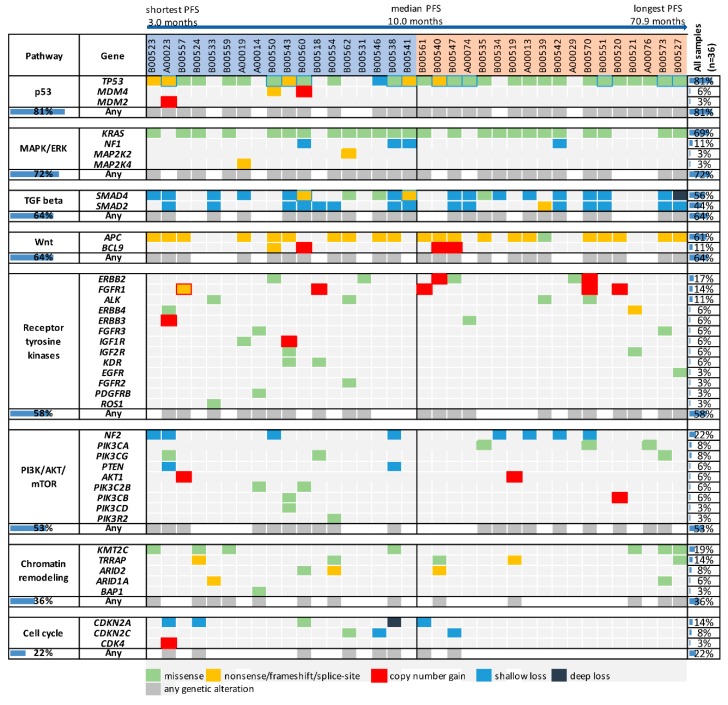
Genetic alterations in oncogenic pathways and frequently mutated genes in bevacizumab-treated patients according to progression-free survival (PFS). Patients were sorted according to their PFS duration, with all patients except the two patients with the longest PFS eventually relapsing during the study period. Genetic alterations are displayed in an oncoprint plot according to their occurrence in genes of important oncogenic pathways. PFS, progression-free survival.

Genetic alteration analysis, including mutations, as well as copy number alterations, was performed for important cancer signaling pathway genes. We then analyzed the relationship between genetic alterations and PFS. An oncoprint plot was created, sorting patients according to their PFS (Figure 1). This sorting approach was feasible in our cohort, since all patients except the two patients with the longest PFS (B00573 and B00527) relapsed during the observation period, preventing the display of censored patients with a short follow-up at inappropriate ranks.

Among the investigated genes, *TP53* was the most frequently altered gene (81%, n = 29). PFS was not different among patients with and without *TP53* alterations, although there was a trend towards longer PFS in patients with *TP53* alterations (HR, 0.58; 95% CI, 0.19–1.37; *p* = 0.188, Table 1). However, patients with truncating mutations seemed to have a shorter PFS compared to patients with missense mutations (Figure 1). *TP53* mutation subtypes may have different biological impacts, since truncating mutations disrupt protein function, whereas missense mutations may be associated with an oncogenic gain-of-function [16]. It is therefore possible that analyzing alterations with discrimination of the mutation subtype may be necessary for this gene. Of note, *TP53* deletions were observed both in patients with missense and truncating alterations.

Among other genes altered in at least five patients (*KRAS, APC, SMAD4, SMAD2, NF2, KMT2C, ERBB2, FGFR1, TRRAP* and *CDKN2A*), only *CDKN2A* alterations were significantly associated with PFS. Patients with *CDKN2A* alterations had shorter PFS than patients without such alterations (HR, 2.93; 95% CI, 1.44–27.10; *p* = 0.017, Table 1). However, *CDKN2A* is located in chromosomal vicinity to *PTPRD*, and deleterious alterations in *PTPRT* and *PTPRD* are associated with treatment resistance in our cohort [13]. Among the five patients with *CDKN2A* shallow loss, three patients (A00023, B00524 and B00561) had losses of both *PTPRD* and *CDKN2A*, including the two *CDKN2A*-altered patients with the shortest PFS (A00023, PFS = 3.1 months, and B00524, PFS = 3.3 months). The remaining two patients with *CDKN2A* alterations (B00538 and B00560) did not have particularly short PFS compared to the median PFS in our cohort (9.2 and 9.7 months, respectively, versus 10.0 months).

Regarding the investigated pathways (p53, MAPK/ERK, TGFβ, Wnt, receptor tyrosine kinases, PI3K/AKT/mTOR, chromatin remodeling, and cell cycle), statistically significant differences in PFS were only observed for the cell cycle (Table 1). However, observed differences were mostly due to *CDKN2A* alterations, and patients without *CDKN2A* alterations, but alterations in other cell cycle genes had PFS durations of 9.5, 9.7 and 12.4 months, respectively. Therefore, no promising new markers of PFS could be identified in our pathway analysis. However, alterations in the JAK/STAT pathway are not included in the graph, since those were previously identified as markers of response, with a simultaneous association with PFS [13].

In summary, the analysis of overall genetic alterations in frequently altered genes or the investigated oncogenic pathways failed to identify new promising biomarkers for PFS. However, for the gene *TP53*, different biological impacts of mutation subtypes may justify a more refined analysis for this gene. In contrast, an alteration subtype analysis did not seem suitable for other genes. *KRAS* mutations were activating codon 12 or 13 mutations in 23 of 25 patients, *APC* harbored truncating mutations in 21 of 22 patients, and *SMAD2* and *SMAD4* were characterized by inactivating mutations or single copy number losses. Alterations in *NF2* and *KMT2C* were restricted to copy number losses and missense mutations, respectively, and *ERBB2*, *FGFR1, TRRAP* and *CDKN2A* were only altered in five to six patients.

### 2.3. TP53 Mutations Detected in the Study Cohort

**Figure 2:**
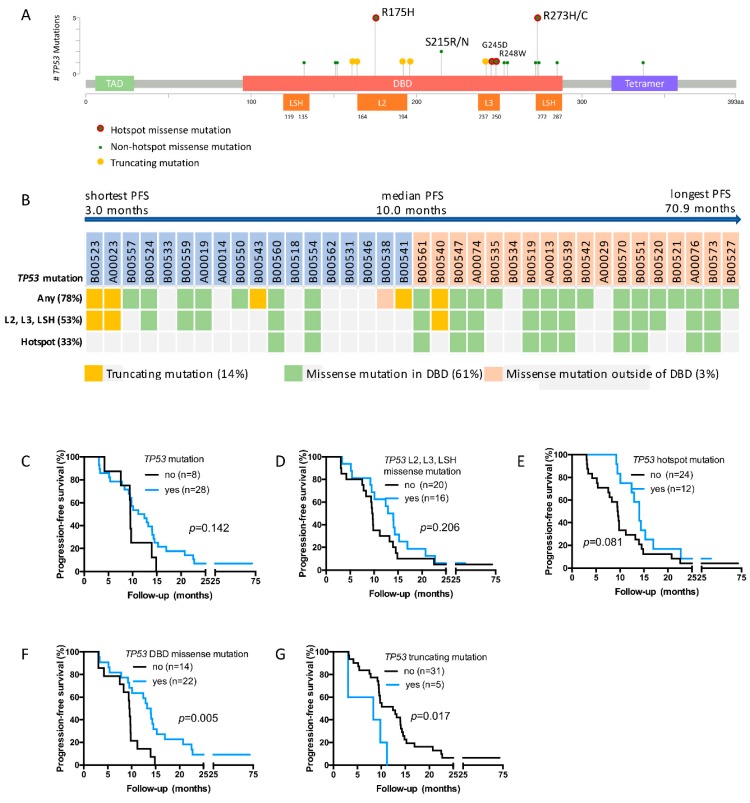
*TP53* mutations and PFS. *TP53* mutations detected in the present study are depicted according to their protein location (**A**). Patients were categorized according to their *TP5*3 mutation characteristics and sorted according to their PFS duration, with all patients except the two patients with the longest PFS eventually relapsing during the study period (**B**). PFS is displayed in Kaplan-Meier plots for patients regarding the occurrence of any *TP53* mutations (**C**), *TP53* L2, L3, LSH missense mutations (**D**), *TP53* hotspot mutations (**E**), *TP53* binding domain (DBD) missense mutations (**F**) and *TP53* truncating mutations (**G**). Statistical analysis was performed by the log-rank test. PFS, progression-free survival.

In the vast majority of the 29 patients with *TP53* alterations, *TP53* mutations were detected (97%, n = 28). The overall *TP53* mutation frequency in our cohort was 78%, and no patient harbored more than one *TP53* mutation. All *TP53* mutations for our cohort are listed in Supplementary Table S2, together with mutation classification and associated clinical data. Mutations are displayed according to their amino acid position in Figure 2A, and were categorized into mutation subtypes as described in the Methods. Truncating mutations were detected in 14% of patients (n = 5). The remaining patients with mutations harbored missense mutations, with all of them except for one being located in the DBD (61%, n = 22). Subtypes of DBD missense mutations were hotspot mutations in 33% (n = 12) of patients, and L2, L3 and LSH missense mutations in 44% (n = 16) of patients. Both of the latter two mutation categories included the most prominent hotspots of our cohort, R175H and R273C/H (each n = 5).

*TP53* mutations in the TCGA cohort were slightly less common than in our cohort, and 123 mutations were detected in 54% (n = 121) of samples. As in our cohort, missense mutations were the most common mutation subtype (n = 84). The majority of those mutations occurred in the DBD, and the most common hotspots were R175H/C (n = 17) and R273H/C (n = 11) (Supplementary Figure S1).

### 2.4. TP53 DBD Missense Mutations Are Associated with Prolonged PFS, Whereas Truncating Mutations Are Associated with Short PFS

We next analyzed PFS in patients harboring different *TP53* mutation subtypes. The median PFS in the overall cohort was 10.0 months (Table 2, Figure 2B). In patients with any *TP53* mutation, L2, L3 and LSH mutations or *TP53* hotspot mutations, median PFS was longer (11.8, 13.5 and 14.0 months, respectively), however differences to the rest of the cohort were not statistically significant (Table 2 and Figure 2C–E). In contrast, patients with *TP53* DBD missense mutations had prolonged PFS compared to patients without such mutations, the median PFS being 13.6 months versus 9.5 months (HR, 0.41; 95% CI, 0.13–0.65; *p* = 0.005; Table 2 and Figure 2F). PFS did not differ in patients with the most common *TP53* DBD missense mutations, R175H and R273C/H (Supplementary Figure S2). In spite of the low number of patients with *TP53* truncating mutations, there was a clear association with short PFS compared to patients without *TP53* truncating mutations (HR, 2.95; 95% CI, 1.45–27.50; *p* = 0.017, Table 2 and Figure 2G).

### 2.5. TP53 DBD Missense Mutations Are an Independent Predictor of PFS

Due to their association with PFS, *TP53* DBD missense mutations and *TP53* truncating mutations were further considered as markers of PFS for bevacizumab-treated patients. *TP53* DBD missense mutations were not associated with any other analyzed clinical characteristics, while *TP53* truncating mutations were associated with rectal tumors (*p* = 0.008, Supplementary Table S3).

We have previously shown that when considering clinical factors and *PTPRT* and *PTPRD* deleterious alterations, rectal tumors and *PTPRT* and *PTPRD* deleterious alterations were independent predictors of short PFS. We therefore performed a multivariate analysis considering *TP53* DBD missense mutations, *TP53* truncating mutations, *PTPRT* and *PTPRD* deleterious alterations, and tumor location as PFS predictors. (Table 3). In multivariate analysis, *TP53* DBD missense mutations retained statistical significance as an independent PFS predictor (HR, 0.31; 95% CI, 0.13–0.77; *p* = 0.011, Table 3), together with tumor location and *PTPRT/PTPRD* deleterious alterations.

## 3. Discussion

Although bevacizumab is a standard first line therapy for metastatic colorectal cancer (5–8), there are still no clinical predictive biomarkers for its use. We previously found an association of deleterious alterations in the genes *PTPRT/PTPRD* of the JAK/STAT pathway with bevacizumab response status [13]. To extend or analysis beyond response, we correlated the results of comprehensive genomic profiling in our cohort with PFS and found an impact of *TP53* mutation subtype on the clinical outcome of bevacizumab therapy in mCRC.

In our study cohort, when considering overall genetic alterations, the only gene significantly correlated with PFS was *CDKN2A*, with copy number losses predominating. The only pathway correlated with PFS was the cell cycle, an association mostly driven by *CDKN2A* alterations. However, *CDKN2A* is located near *PTPRD* on chromosome 9, and its loss was associated with *PTPRD* loss. Due to this association, *CDKN2A* alterations were not considered as a new independent marker for bevacizumab treatment outcome. Results from the literature do not show survival differences in bevacizumab-treated CRC patients according to *KRAS* mutation status [10,11,17]. Similarly, *TP53* mutation status was not associated with survival in bevacizumab-treated patients [11]. Our results are in line with those studies, although there was a tendency for prolonged PFS for patients with overall *TP53* mutations (HR, 0.57; 95% CI, 0.19–1.24; *p* = 0.142). When comparing anti-angiogenic regimens to non-anti-angiogenic regimens, patients with *TP53* mutations had a higher treatment benefit than *TP5*3 wild-type patients in diverse cancer types [18,19]. Associations were less clear for CRC patients [11,18], however the discrepant results between CRC and other cancers might be partly related to study design and a different prevalence or impact of functionally different *TP53* mutation subtypes.

Some CRC studies have found *TP53* mutations in about 55% of patients [20,21]. However, the *TP53* mutation rate in non-hypermutated tumors is slightly higher and was about 60% in the TCGA study [20]. Most importantly, *TP53* mutations are enriched in tumors from patients with metastatic disease [22]. In the MSK cohort, *TP53* mutation frequencies were 78% for MSS patients, and ranged between 74% and 80% for patients with metastatic disease [22]. In our study, we enrolled patients with metastatic disease, and all patients had MSS tumors. Therefore, the observed *TP53* mutation rate of 78% is within the expected range. Due to the high mutation rate, mutation subtype analysis was feasible, despite our relatively moderate cohort size (n = 36). No association with response was found for any *TP53* mutation subtypes. However, PFS analysis revealed that *TP53* DBD missense mutations conferred a statistically significant and clinically meaningful survival benefit of bevacizumab therapy in mCRC patients (HR, 0.41; 95% CI, 0.13–0.65; *p* = 0.005). In contrast, *TP53* truncating mutations were associated with short survival (HR, 2.95; 95% CI, 1.45–27.50; *p*=0.017), although it should be noted that the number of patients with *TP53* truncating alterations was extremely small (n = 5). In multivariate analysis of PFS, *TP53* DBD missense mutations were an independent factor, together with *PTPRT/PTPRD* deleterious alterations and rectal tumor site. Although positive associations of *T53* DBD missense mutations and survival could be prognostic and not predictive, however, the literature does currently not support this notion. *TP53* missense mutations were associated with lower survival rates in CRC patients, at least in univariate analysis [23]. Similarly, *TP53* DBD missense mutations are a negative prognostic factor for survival in breast cancer and oral cavity squamous cell carcinoma [24,25].

Wild type p53 was found to repress the VEGF activity via interaction with several transcription factors, including SP1 and E2F [26,27], and *TP53* involvement in angiogenic processes may be considered to explain the observed association with bevacizumab treatment outcome. A recent study showed that *TP53* mutation correlated with upregulation of VEGF-A in NSCLC [28]. However, while VEGF-A upregulation has been suggested to be responsible for a better anti-angiogenic treatment outcome in that study, enhanced VEGF-A expression was discussed as a resistance mechanism in CRC [29]. While underlying mechanisms may be complex and are not yet fully understood, it appears reasonable that different *TP53* mutation subtypes may have different impacts on angiogenesis and bevacizumab treatment outcome.

*TP53* mutations may have different functional consequences [30]: loss of function [31], selection of function [20] and gain of function [32]. A gain of function means a gain of oncogenic function such as promoting cell proliferation, anti-apoptosis, migration, invasion, angiogenesis, and metastasis [33,34,35]. While truncating *TP53* mutations likely result in a loss of wild-type protein function, many *TP53* DBD missense mutations may lead to the acquisition of new functions, e.g., by p 53 binding to transcription factors and modulation of target gene expression [20,36], and certain *TP53* DBD missense mutations have been suggested to promote angiogenesis [37]. In this context, it would be interesting to perform gene expression profiling to analyze whether the presence of *TP5*3 DBD missense mutations is related to changes in gene expression that could explain an association with bevacizumab treatment outcome. Such experiments could further be designed to evaluate a possible impact of altered interactions between mutant p53 and other transcription factors.

Using *TP53* DBD missense mutations for patient stratification appears promising considering the relatively high difference in PFS between marker-positive and negative patients (HR, 0.41) [38]. However, due to the small sample size in our study, it is essential to validate our results in larger patient cohorts. Another limitation of our study is its retrospective nature, and the findings should be confirmed in prospective studies.

## 4. Methods

### 4.1. Patients, Treatment and Next-Generation Sequencing

Study approval and written informed consent were provided by the Institutional Review Board at the Chang Gung Memorial Hospital (IRB 102-2850A3) and all included patients, respectively. Patient enrollment criteria, details regarding the next-generation sequencing of tumor samples, and sequence and copy number variant identification have described in a previous study of the same patient cohort [13].

### 4.2. Variant Classification

Shallow copy number losses were defined as an observed copy number < 2 with SNP analysis indicating a loss of heterozygosity (LOH) in the gene of interest. Analyzed SNPs included those present in the gene of interest or within the chromosomal vicinity within or beyond the cytoband harboring the gene of interest. If the gene of interest had no suitable SNPs, SNPs on both sides of the gene needed to indicate an LOH. Deep losses were defined as observed copy numbers ≤ 1 without apparent LOH. Copy number gains were defined as observed copy numbers ≥ 3 with copy numbers in the tumor being ≥ 3.5.

Copy number variants could be included the in functional analysis if they were copy number losses of genes inactivating investigated oncogenic pathways or gains of genes activating those pathways. Investigated genes for which copy numbers < 2 or gains were observed in our study patients, as well their inclusion in copy number analysis, are listed in Supplementary Table S1.

*TP53* mutations were classified as (1) any *TP53* mutations, (2) *TP5*3 truncating mutations, namely frameshift, nonsense and splice site variants, (3) *TP53* DNA binding domain (DBD) missense mutations, namely missense variants at amino acids 95-289, (4) *TP53* hotspot missense variants, namely at positions R175, Y220, G245, R248, R273, R282 [39,40], and (5) variants in L2, L3 and LSH, namely mutations occurring at the L2 amino acid positions 164–194, the L3 positions 237–250 or the LSH positions 119–135/272–287. The graphical visualization of results was based on tools provided by cBioPortal [41,42].

### 4.3. Statistical Analysis

The statistical evaluation of study results was performed with GraphPad Prism, v. 6.0 and SPSS, v. 20.0.0. Used statistical tests were the Fisher’s Exact tests for categorial outcomes, the Mann-Whitney test for tumor change from baseline and the log-rank test and Cox regression for survival outcomes. For multivariate analysis, Cox regression was performed using the “Enter” method (SPSS).

## 5. Conclusions

The present study demonstrated the clinical utility of targeted next-generation sequencing in mCRC patients to identify genetic biomarkers for anti-angiogenic therapy. To our knowledge, this is the first study to suggest *TP53* DBD missense mutations as a biomarker of sensitivity to bevacizumab treatment in mCRC. However, its predictive value warrants further confirmation in clinical studies.

## Figures and Tables

**Table 1:** Statistical analysis of the association of progression-free survival (PFS) with frequently altered genes and cancer signaling pathways.

	Genetic Alterations	n (%)	Median PFS (Months)	HR (95% CI)	*p* Value
**All patients**		36 (100)	10.0		
Gene					
*TP53*	no	7 (19)	9.5	1.00	0.188
	yes	29 (81)	11.2	0.58 (0.19–1.37)	
*KRAS*	no	11 (31)	11.2	1.00	0.863
	yes	25 (69)	9.8	1.06 (0.52–2.17)	
*APC*	no	14 (39)	9.7	1.00	0.645
	yes	22 (61)	10.7	0.85 (0.42–1.70)	
*SMAD4*	no	16 (44)	9.8	1.00	0.728
	yes	20 (56)	11.1	0.89 (0.45–1.74)	
*SMAD2*	no	20 (56)	9.9	1.00	0.375
	yes	16 (44)	11.1	0.74 (0.38–1.44)	
*NF2*	no	28 (78)	10.0	1.00	0.502
	yes	8 (22)	11.9	1.30 (0.57–3.17)	
*KMT2C*	no	29 (81)	10.1	1.00	0.125
	yes	7 (19)	9.8	0.51 (0.24–1.13)	
*ERBB2*	no	30 (83)	9.8	1.00	0.915
	yes	6 (17)	11.8	1.05 (0.43–2.57)	
*FGFR1*	no	31 (86)	9.8	1.00	0.986
	yes	5 (14)	10.1	0.99 (0.38–2.56)	
*TRRAP*	no	31 (86)	9.8	1.00	0.571
	yes	5 (14)	11.2	0.74 (0.30–1.94)	
*CDKN2A*	no	31 (86)	12.4	1.00	0.017
	yes	5 (14)	9.2	2.93 (1.44–27.10)	
**Pathway**					
p53	no	7 (19)	9.5	1.00	0.188
	yes	29 (81)	11.2	0.58 (0.19–1.34)	
MAPK/ERK	no	10 (28)	12.1	1.00	0.642
	yes	26 (72)	9.8	1.19 (0.58–2.43)	
TGFβ	no	13 (36)	10.1	1.00	0.947
	yes	23 (64)	9.8	0.98 (0.49–1.95)	
Wnt	no	13 (36)	9.8	1.00	0.815
	yes	23 (64)	10.1	0.92 (0.46–1.85)	
Receptor tyrosine kinases	no	15 (42)	9.8	1.00	0.594
	yes	21 (58)	10.1	0.83 (0.42–1.65)	
PI3K/AKT/mTOR	no	17 (47)	10.1	1.00	0.981
	yes	19 (53)	9.8	1.01 (0.51–1.98)	
Chromatin remodeling	no	23 (64)	12.4	1.00	0.771
	yes	13 (36)	9.5	0.90 (0.44–1.82)	
Cell cycle	no	28 (78)	13.1	1.00	0.017
	yes	8 (22)	9.6	2.44 (1.35–11.40)	

Statistical calculations were performed using the log-rank test. CI, confidence interval; HR, hazard ratio; PFS, progression-free survival.

**Table 2:** *TP53* mutation subtypes and clinical outcome.

	n (%)	Median PFS (Months)	HR (95% CI)	*p* Value	Responders n (%)	*p* Value	Median Tumor Change from Baseline	*p* Value
**All patients**	36 (100)	10.0			18 (50)		−25%	
*TP53* mutation								
no	8 (22)	9.5	1.00	0.142	4 (50)	1.000	−25%	0.759
yes	28 (78)	11.8	0.57 (0.19–1.24)		14 (50)		−25%	
*TP53* L2, L3, LSH missense mutation								
no	20 (56)	9.6	1	0.206	9 (45)	0.738	−17%	0.260
yes	16 (44)	13.5	0.65 (0.33–1.27)		9 (56)		−32%	
*TP53* hotspot missense mutation								
no	24 (67)	9.5	1	0.081	10 (42)	0.289	−15%	0.029
yes	12 (33)	14.0	0.54 (0.28–1.07)		8 (67)		−35%	
*TP53* DBD missense mutation								
no	14 (39)	9.5	1.00	0.005	6 (43)	0.733	−17%	0.482
yes	22 (61)	13.6	0.41 (0.13–0.65)		12 (55)		−30%	
*TP53* truncating mutation								
no	31 (86)	12.4	1.00	0.017	16 (52)	1.000	−30%	0.761
yes	5 (14)	8.3	2.95 (1.45–27.50)		2 (40)		−16%	

Statistical calculations were performed using the log-rank test, Fisher’s Exact test and Mann-Whitney test, respectively. CI, confidence interval; HR, hazard ratio.

**Table 3:** Multivariate analysis of Progression-free survival (PFS).

Factors	n	HR (95% CI)	*p* Value
Clinical			
Primary tumor site (rectum/colon)	15/21	2.36 (1.05–5.31)	0.037
Genetic			
*TP53* truncating mutations (yes/no)	5/31	1.26 (0.35–4.55)	0.725
*TP53* DBD missense mutations (yes/no)	22/14	0.31 (0.13–0.77)	0.011
*PTPRT/PTPRD* deleterious alteration (yes/no)	10/26	3.87 (1.66–9.00)	0.002

Multivariate analysis was performed by Cox regression, considering tumor location, *TP53* DBD missense mutations, *TP53* truncating mutations and *PTPRT/PTPRD* deleterious alterations as predictive factors for PFS.

## Supplementary Materials

The following are available online at https://www.mdpi.com/2072-6694/11/8/1079/s1. Table S1: Gene selection for copy number analysis. Genes analyzed in this study for which patients had copy number gains are listed, and genes for which these gains were included in the genetic alterations correlated with PFS are indicated. Additionally, genes for which patients had observed copy numbers <2 are listed. Genes for which copy number loss was further considered for analysis of shallow loss/deep loss and correlation with PFS are indicated. PFS, progression-free survival; Table S2: *TP53* mutations and clinical outcome for all study patients. Data of clinical outcome and results of *TP53* mutation analysis, including mutation categories for detected *TP53* mutations, are listed for all study patients. PFS, progression-free survival; Table S3: Clinicopathological characteristics of patients with *TP53* DBD missense and *TP53* truncating mutations. Patient characteristics were analyzed for patients with *TP53* DBD missense mutations versus patients with wild-type *TP53* or *TP53* non-DBD missense mutations. Additionally, patient characteristics were compared for patients with *TP53* truncating mutations versus patients with wild-type *TP53* or *TP53* non-truncating mutations. Statistical calculations were performed using the Fisher’s Exact test; Figure S1: *TP53* mutations in the TCGA study. A graph displaying all *TP53* mutations detected in the TCGA colorectal cancer patient cohort (n = 224) according to their amino acid position is shown; Figure S2: PFS in patients with *TP53* R175H and R273H/C mutations. PFS was analyzed for patients with *TP53* R175H and R273H/C mutations (each n = 5). Statistical calculations were performed using the log-rank test. PFS, progression-free survival.
