# Supplementary figures and images for: TP53 DNA Binding Domain Mutations Predict Progression-Free Survival of Bevacizumab Therapy in Metastatic Colorectal Cancer
Source: Cancers (Basel). 2019 Jul 30;11(8):1079. doi: 10.3390/cancers11081079 (PMC6721375; doi:10.3390/cancers11081079)

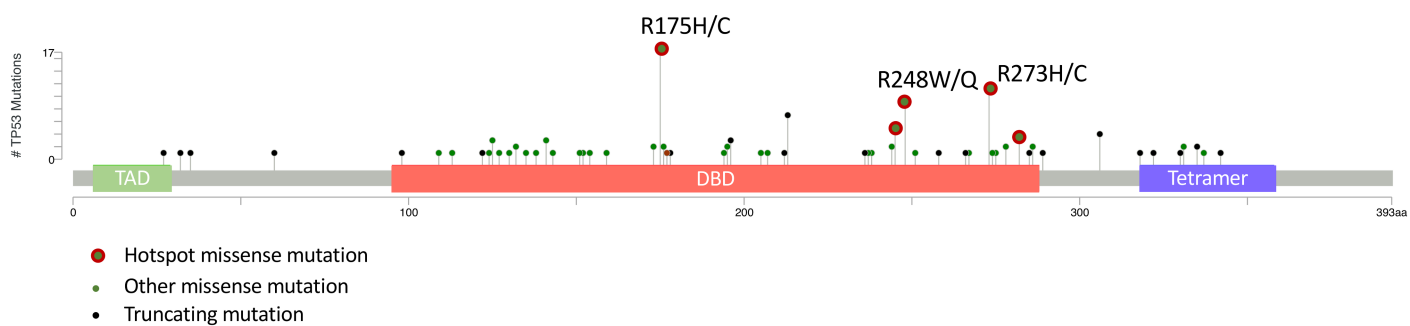

Supplement: Supplementary file 1 [file cancers-11-01079-s001.zip › Supplementary Figure 1.pdf]

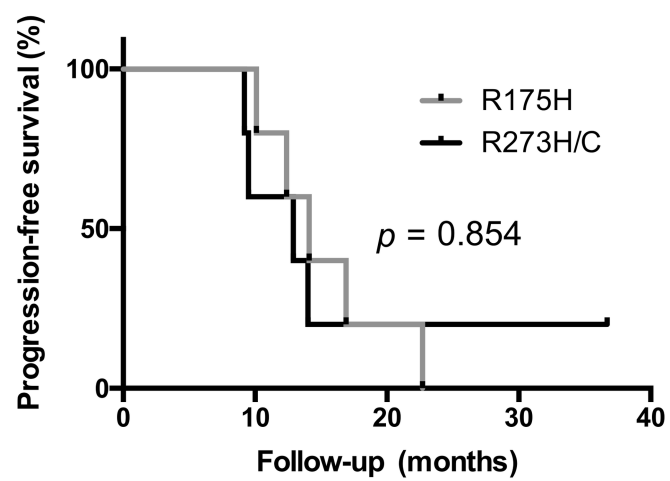

Supplement: Supplementary file 1 [file cancers-11-01079-s001.zip › Supplementary Figure 2.pdf]
